# Neither Lys- and DAP-type peptidoglycans stimulate mouse or human innate immune cells via Toll-like receptor 2

**DOI:** 10.1371/journal.pone.0193207

**Published:** 2018-02-23

**Authors:** Marybeth Langer, Alanson W. Girton, Narcis I. Popescu, Tarea Burgett, Jordan P. Metcalf, K. Mark Coggeshall

**Affiliations:** 1 The Oklahoma Medical Research Foundation, Program in Arthritis & Immunology, Oklahoma City, OK, United States of America; 2 The Department of Microbiology and Immunology, University of Oklahoma Health Sciences Center, Oklahoma City, OK, United States of America; 3 The Department of Medicine, Pulmonary and Critical Care Div., University of Oklahoma Health Sciences Center, Oklahoma City, OK, United States of America; Pusan National University, REPUBLIC OF KOREA

## Abstract

Peptidoglycan (PGN), a major component of bacterial cell walls, is a pathogen-associated molecular pattern (PAMP) that causes innate immune cells to produce inflammatory cytokines that escalate the host response during infection. In order to better understand the role of PGN in infection, we wanted to gain insight into the cellular receptor for PGN. Although the receptor was initially identified as Toll-like receptor 2 (TLR2), this receptor has remained controversial and other PGN receptors have been reported. We produced PGN from live cultures of *Bacillus anthracis* and *Staphylococcus aureus* and tested samples of PGN isolated during the purification process to determine at what point TLR2 activity was removed, if at all. Our results indicate that although live *B*. *anthracis* and *S*. *aureus* express abundant TLR2 ligands, highly-purified PGN from either bacterial source is not recognized by TLR2.

## Introduction

During bacterial sepsis, the bacterial-produced pathogen-associated molecular patterns (PAMPs) are responsible for activation of the complement and coagulation systems, contributing to organ failure and death of the host [1, 2]. Peptidoglycans (PGN), a disaccharide polymer with peptide cross-linkers and present in bacterial cell walls, have been established as important bacterial PAMPs: PGN has been shown to induce inflammatory mediators from human innate immune cells, to stimulate platelet aggregation and prothrombinase activity, and to stimulate complement consumption [3–5]. Animals challenged with PGN show features of sepsis pathology [6], suggesting that PGN is an important PAMP in this condition.

PGN is a large glycan polymer composed of alternating units of N-acetylglucosamine (GlcNac) and N-acetylmuramic acid (MurNac) residues joined by stem peptide units consisting of 4 or 5 L- and D-amino acids [7]. The stem peptides may be linked to each other or they may be joined by interpeptide bridges that have a different amino acid composition than the stem peptides. The stem peptides and bridges vary between species in their length and composition. For example, *B*. *anthracis* PGN has a stem peptide consisting of three amino acids including diaminopimelic acid (DAP) [3], while *S*. *aureus* contains a lysine residue in the DAP position and has a pentapeptide stem linked by a pentaglycyl bridge [8]. The relevance of these differences in the proinflammatory properties of PGN in mammals has not been studied.

In order to understand the role of PGN during the pathophysiology of bacterial infections, it is important to know the cellular receptor(s) that binds PGN. The PGN receptor is responsible for initiation of the inflammatory response and could contribute to coagulopathy in sepsis. We reported that human monocytes and neutrophils produce inflammatory cytokines [3, 5, 9] and that platelets exhibit a procoagulant state [4] in response to PGN. The PGN receptor on monocytes was initially reported to be a pattern recognition receptor (PRR), specifically Toll-like receptor 2 (TLR2) [10, 11]. However, it has also been reported that highly purified PGN does not stimulate through TLR2, or that other receptors may also play a prominent role [12]. Since PGN is purified from Gram-positive bacterial cell walls, preparations of PGN may contain cell wall impurities such as lipoteichoic acids (LTA), wall teichoic acids (WTA), lipoproteins and glycolipids. These impurities can all stimulate an inflammatory response in immune cells, and may be the reported stimulant in PGN preparations.

Alternative means of PGN recognition have been described. We reported evidence for IgG/Fc receptors (FcγR) as an important receptor that indirectly recognizes PGN [3, 9, 13]. We showed that normal human plasma contains IgG that opsonizes PGN, that plasma or anti-PGN antibodies were required for PGN binding to monocytes and neutrophils and that PGN was bound and phagocytosed by HEK293 cells transfected with FcγRIIA in the presence of human plasma. These results suggested a model in which polymeric PGN is opsonized by IgG, allowing FcγR internalization, trafficking to lysosomes, and lysosomal degradation to NOD ligands [3, 9, 13].

We hypothesized that the conflicting data on PGN as a TLR2 ligand may either be due to differences in purification procedures where other cell wall components might contaminate preparations or due to intrinsic structural differences within PGN from different bacterial species. Here, we purified PGN of the Lys-type from *S*. *aureus* and of the DAP-type from *B*. *anthracis*. We compared the TLR2 induction activity throughout the steps of the purification process to determine whether any steps had a significant influence on the amount of TLR2 ligands in the sample. We found that *B*. *anthracis* and *S*. *aureus* bacteria contain TLR2 ligands, but purified PGN from either source is not a TLR2 ligand. We also found that *S*. *aureus* PGN contains palmitoylated peptide(s) that cannot be removed by our PGN purification procedures.

## Materials and methods

### Materials

Brefeldin A, anti-human TNFα-Allophycocyanin (APC; clone MAb11), isotype control mouse IgG1-APC (clone P3.6.2.8.1), anti-human CD14-PeCy7 (clone 61D3), anti-mouse CD11b-PE (clone M1/70), rat IgG2b K isotype control (clone eB149/10H5), anti-mouse TNFα-APC (clone MP6-T22) and rat IgG1 K Isotype Control-APC (clone eBRG1) were purchased from eBioscience. Cytochalasin D was purchased from Sigma. Low IgG fetal bovine sera (FBS) was purchased from Gibco and used throughout. Human IgG was obtained from Talecris (Immune Globulin Human GamaSTAN S/D). Proteinase K (≥20 U/mg) was obtained from Life Technologies. *S*. *aureus* strain MN8 was from BEI Resources and *B*. *anthracis* strain delta Stern was obtained from S. Kurosawa, Boston University School of Medicine, Boston MA. The Newman and ∆*lgt* strains of *S*. *aureus* were provided by Dr. Dominique Missiakas of the University of Chicago, Division of Biological Sciences.

### Ethics statement

Studies on primary human cells and blood products were performed according to protocols approved by the Oklahoma Medical Research Foundation Institutional Review. All animal studies were approved by the Oklahoma Medical Research Foundation Institutional Animal Care and Use Committee.

### PGN preparation

Non-pyrogenic plastic labware was used throughout the procedure after harvesting the bacteria. Endotoxin-free water was used throughout for all media, solutions and washes with water. Centrifugations were at 15,000g for 10 min at room temperature, unless otherwise stated. *B*. *anthracis* Delta Sterne strain was grown to stationary phase in 4–500 ml cultures of Trypticase^TM^ Soy Broth (BD Biosciences). The cultures were harvested by centrifugation at 4°C and washed once in water. The bacterial pellets were combined in eight 50 ml polysulfone Oak Ridge centrifuge tubes and resuspended in 30 ml 8% SDS, boiled for 30 min, cooled, centrifuged and the process was repeated. After the second SDS extraction, the cell wall extract was centrifuged and the pellets were washed 4 times in 30 ml water to remove the SDS. The pellets were resuspended in 6 mls water. Tryptic soy agar plates were streaked with 10 μl of the preparation and incubated for 24 hours at 37°C to ensure there were no viable cells before continuing the procedure. The cell wall extract was digested with DNase and RNase by resuspending each pellet in 3 ml of solution containing 331 U/ml of DNase 1 (Invitrogen), 42 U/ml RNase A and 1667 U/ml RNase T1 (Ambion RNase cocktail) in DNase 1 buffer (10 mM Tris base, 0.5 mM CaCl_2_, 2.5 mM MgCl_2_, pH 7.5) and incubating with rocking for 30 min at room temperature and centrifuged. The DNase/RNase digestion was repeated, the material was centrifuged and boiled in SDS solution as above to remove the digestive enzymes. The material was centrifuged and washed repeatedly (4–5 times) with water. After the last wash, the material was resuspend in 30 ml ice cold 48% hydrofluoric acid (HF), vortexed, incubated with rocking for 6 hours at 4°C and centrifuged. The pellets were resuspended in 30 ml of ice cold HF, incubated overnight with rocking at 4°C and centrifuged. The extract was centrifuged and washed four times with water. The pellet was resuspended in denaturing buffer (50 mM Tris base, 6M guanidine HCl, 25 mM dithiothreitol, pH 8) and incubated for 1 h at 60°C. The extract was cooled to room temperature and the denaturing buffer was removed by centrifugation. The pellet was incubated in 0.8 M Iodoacetamide, 0.8M Tris base, pH 8 in the dark for 15 min at room temperature and then washed with water. We have prepared PGN including denaturation and alkylation by iodoacetamide or not including these steps. The final products are identical in biological activity. Our current PGN purification protocol does not include denaturation and alkylation.

The extract was resuspended in 30 ml proteinase K buffer (50 mM Tris base, 1 M guanidine HCl, 5 mM CaCl_2_, pH 7.5), and proteinase K was added to 0.67 mg/ml to each tube and incubated at 50°C on an orbital shaker overnight. The digested material was centrifuged and washed with water 3–4 times. The PGN was sonicated using the Misonix Ultrasonic Liquid Processor (Sonicator S-400) set at amplitude 50 with two 2.5 min pulses, separated by one minute without pulse. After sonication, the PGN was pooled, evenly distributed in Oakridge tubes, centrifuged, and resuspended in 30 ml 8% SDS. The now pure PGN was boiled for 30 min, cooled, washed with water and repeated. The PGN suspension was transferred to several pre-weighed endotoxin free microfuge tubes for drying. The suspension was dried in a speed vacuum and the microfuge tubes were weighed to obtain the weight of the PGN. The PGN was resuspend at a concentration of 10 mg/ml in water. The phosphate content of PGN preparations was measured as described [14]. The PGN was stored at -20°C for long-term storage or 4°C for short-term storage.

### Assay of TLR2 and TLR4 activity of PGN

TLR2 and TLR4 activity assays were performed with reporter HEK293 cells, stably expressing human TLR2 and CD14 (HEK-TLR2), or human TLR4 and MD-2/CD14 (HEK-TRL4), both from Invivogen. Both cell lines also stably express a secreted embryonic alkaline phosphatase (SEAP) reporter. The SEAP reporter gene is under the control of the IFNβ minimal promoter fused to five NF-κB and AP-1-binding sites. SEAP is produced when the specific TLR receptor is stimulated by its ligand, through activation of NF-kB and AP-1. Levels of SEAP are determined by spectrophotometrically at 630 nm after chromogenic conversion of the HEK-Blue Detection Medium (Invivogen). The cells were grown to 80–90% confluence in complete Dulbecco's modified Eagle's medium (Gibco) supplemented with 10% heat-inactivated FBS, penicillin (1,000 units/ml) and (streptomycin 100 μg/ml), 2 mM GlutaMAX (L-alanyl-L-glutamine dipeptide; Gibco) HEK-Blue selection (InvivoGen), and 100 μg/ml Normocin (InvivoGen), at 37°C in a 5% saturated CO_2_ atmosphere. To perform the assay, the cells were resuspended in HEK-Blue Detection Medium and plated in 96 well non-tissue culture treated plates at a concentration of 30–40,000 cells per well for HEK-TLR2, and 50–60,000 cells per well for HEK-TLR4. The cells were incubated with control stimulants, test substances as indicated, vehicle (endotoxin-free water), or media only, at 37°C in a 5% CO_2_ for 16 hours. The optical density at 630 nm was quantified on an Epoch 2 Microplate Reader (BioTek). TLR2 and TLR4 activity was expressed as a fold difference of the OD over the negative control, endotoxin-free water. For TLR4, activity that was lower than that of 10 pg/ml lipopolysaccharide (LPS) was considered nonstimulatory to monocytes as we previously showed [5].

### Assay of biological activity of PGN in human monocytes

Peripheral blood mononuclear cells (PBMC) were prepared from heparinized blood by centrifugation through Histopaque 1077 (Sigma) according to the manufacturers protocol. PBMCs were washed, resupended in RPMI supplemented with 1% FBS and 0.2 mg/ml human IgG, and stimulated for 12 hours in the presence of Brefeldin A (3 μg/ml) with 10 μg/ml PGN of the initial SDS extraction, after HF treatment and PGN after final purification. In some experiments, internalization-dependent signaling was inhibited by pretreatment of PBMCs with cytochalasin D (5 μM; Sigma), a pharmacologic inhibitor of phagocytosis. TNFα production by CD14+ monocytes was quantified by immunostaining after saponin permeabilization using with anti-human CD14-PeCy7 (1 μg/ml) and anti-human TNFα-APC (1 μg/ml) or isotype control, mouse IgG1-APC (1 μg/ml). Data were collected by flow cytometry on a BD LSR II or BD FACSCelesta systems and analyzed using the FlowJo software package (FlowJoLLC).

### A assay of TLR2 activity of PGN in bone marrow-derived mouse macrophages

Mouse strains C57BL/6J (wild type (WT)) and Tlr2 null (B6.129-*Tlr2tm1Kir*/J, Jackson Laboratory) were used for the generation of bone marrow-derived macrophages (BMDM). Femurs were collected from mice and bone marrow was flushed with medium. Bone marrow cells were cultured in RPMI 1640 medium supplemented with 10% FBS, 50 ng/ml recombinant murine monocyte colony stimulation factor-1 (CSF-1, purchased from R&D Systems), and 200 mM Glutamax-I for 24 hours. Non-adherent cells were then harvested, and resuspended in fresh medium, and cultured for 5 additional days in the same media. Macrophages were harvested, re-suspended in culture medium and plated into wells of a 96-well plate overnight at 37°C. Cells were then stimulated for 6 hours with *B*. *anthracis* PGN (10 μg/ml), *S*. *aureus* PGN (10 μg/ml), or LPS (1μg/ml) in the presence of Brefeldin A (3 μg/ml). Following stimulation, the cells were washed in PBS with Brefeldin A and incubated with mouse Fc-block (BD Pharmingen) on ice for 10 minutes. Cells were washed with PBS/Brefeldin A and fixed with 1% formaldehyde. After fixation, cells were permeabilized with 0.5% saponin and then stained with anti-mouse CD11b-PE (2 μg/ml), anti-mouse TNFα-APC (2 μg/ml) or appropriate isotype control (2 μg/ml). Macrophages were identified as CD11b^+^ and TNFα producing cells were quantitated by flow cytometry.

### Amino acid analysis

PGN samples were removed for analysis at the indicated points during the extraction process from *S*. *aureus* and *B*. *anthracis*, after HF treatment, but before Proteinase K digestion. Samples were also taken from the final product that had been digested with Proteinase K, extracted with SDS and washed repeatedly to remove SDS. For the *S*. *aureus* PGN, an additional HF treatment, SDS extraction and washing was performed on the final product and a sample was obtained. The samples were dried and resuspended in water at 10 mg/ml. Amino acid analysis was performed on each sample according to the method described by [15]. Briefly, 1 mg of each PGN sample was lyophilized. 300 μl of 6M HCl with 0.1% phenol was added, the sample was divided into three replicates and the solution was heated to 110°C for 48 hours in order to hydrolyze the peptide bonds. Each sample was cooled to room temperature and vacuum-dried to remove the HCl.

Each sample was resuspended in 10 mM HCl and 20 μl of the sample was added to 60 μl of 0.2M sodium borate buffer, pH 8.8. To this mixture, 20 μl of 6-aminoquinoyl-N-hydroxysuccinimidyl carbamate in acetonitrile was added to derivatize the amino acids present in the sample. The derivatized sample was heated to 55°C for 15 min to convert tyrosine byproducts to one form. High-Performance Liquid Chromatography using an Agilent 1260 series instrument and a Waters AccQ Tag 3.9x150mm column was used to separate and quantify the derivatized amino acids present in each sample. Quantification was accomplished by UV absorbance at 254nm.

### Statistical analysis

Statistical analysis and graphic representation were done using GraphPad Prism (GraphPad Software Inc.). Where applicable, the data are expressed as the mean +/- standard error of the mean. Statistical significance was determined by analysis of variance (ANOVA) with Bonferroni post test. A p value of < 0.05 was considered significant.

## Results

### *B*. *anthracis* and *S*. *aureus* PGN extraction

**Fig 1:**
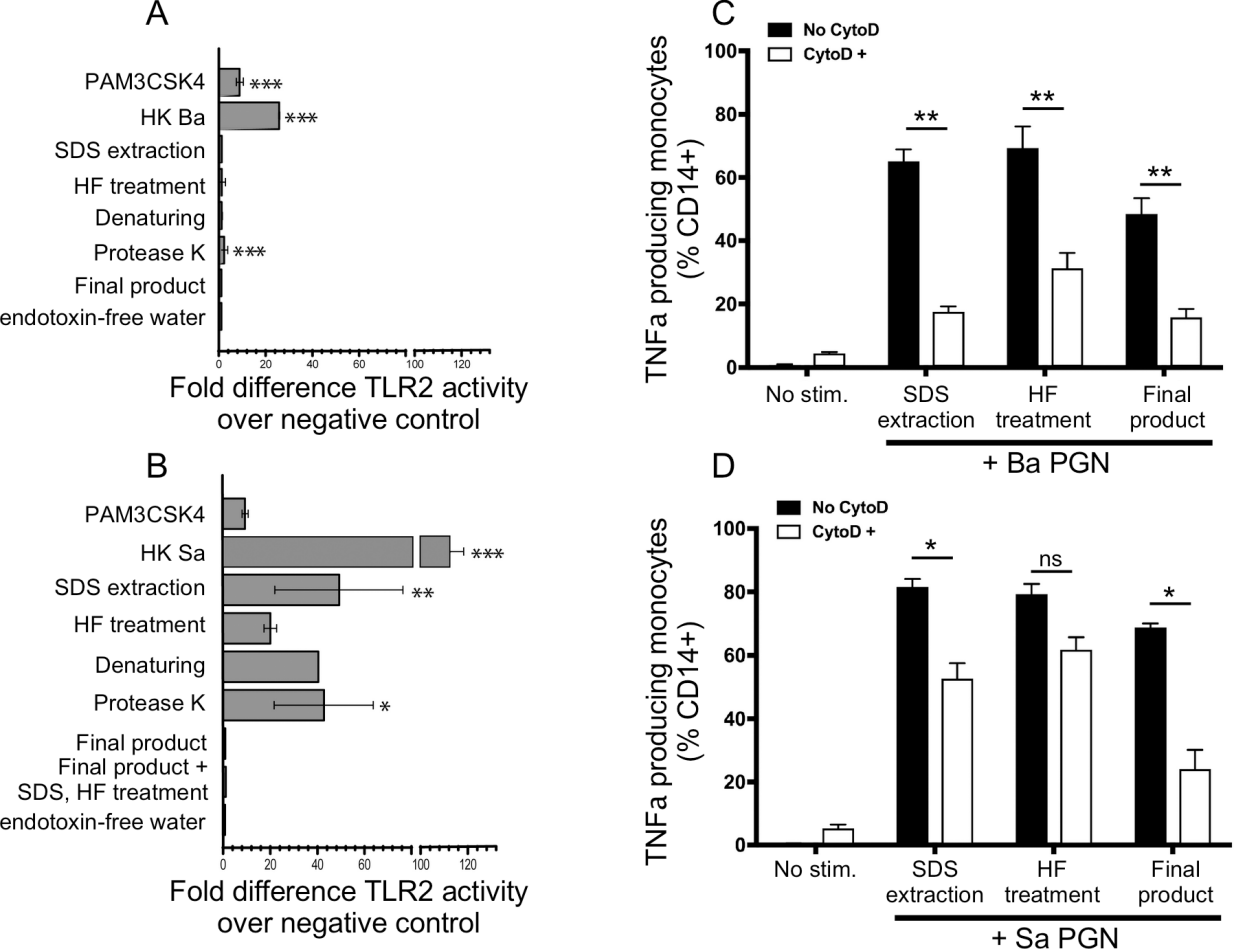
TLR2 activity of *S*. *aureus* and *B*. *anthracis* PGNs. *B*. *anthracis* (A) and *S*. *aureus* (B) PGN samples were taken after the indicated steps of the purification process. The samples were washed three times with endotoxin free (LAL) water, dried, weighed and tested for TLR2 activity at a concentration of 10 μg/ml, using the HEK-TLR2 cell assay. The data are expressed as the mean ± SEM fold difference TLR2 activity over the negative control, endotoxin free water. Statistical significance was determined by ANOVA with Bonferroni post test. *, **p<0.01, ***p<0.001 *versus* endotoxin free water. C & D: Freshly prepared peripheral blood mononuclear cells were stimulated for 12 hours with 10 μg/mL *B*. *anthracis* (Panel C) or *S*. *aureus* (Panel D) PGN samples taken at defined steps during the purification procedure, as described in Panels A & B. The cells were stained for intracellular TNFα and then analyzed by flow cytometry. The internalization requirement was assessed in the presence of 15 μM cytochalasin D (CytoD). Data are expressed as the mean ± SEM of TNFα+CD14+ cells. Statistical significance was determined by paired two-way ANOVA with Bonferroni post hoc test for multiple comparisons. *p<0.05, **p<0.01, ***p<0.001, ns p>0.05.

We extracted DAP- and Lys-type PGN from *B*. *anthracis* and *S*. *aureus*, respectively, and removed samples for analysis at key steps in the procedure. The major steps of the extraction were an initial boiling in 8% SDS to lyse the cells and denature proteins, followed by treatment with DNase and RNase to remove nucleic acids. The preparation was then treated with HF to hydrolyze phosphodiester bonds in LTA and WTA, followed by treatment with proteinase K to remove proteins. Finally, the preparation was boiled again in 8% SDS and washed extensively with endotoxin-free water. We measured TLR2 activity in sample aliquots using HEK293 cells transfected with human TLR2 and a NF-Kb reporter construct. The results for the PGN derived from *B*. *anthracis* (Fig 1A) show significant TLR2 induction activity present in the heat-killed bacteria (HKBA) that was completely removed from the *B*. *anthracis* samples during the initial SDS extraction, compared to the negative control, endotoxin-free water. *S*. *aureus*-derived PGN (Fig 1B) had significant TLR2 activity that became undetectable using the HEK-TLR2 detection system after the proteinase K treatment and final SDS extraction.

In both *S*. *aureus* and *B*. *anthracis*-derived material, the TLR2 activity increased after proteinase K treatment, likely due to introduction of exogenous TLR2 ligands from the recombinant enzyme used in this step. The introduced TLR2 ligands in *B*. *anthracis*-derived PGN was not detectable using the HEK-TLR2 transfectants after the additional purification steps of SDS extractions and water washes. However, *S*. *aureus*-derived material consistently had a very low and statistically-insignificant level of TLR2 inducing activity. We subjected the *S*. *aureus*-derived PGN to an additional SDS extraction and HF treatment and found that the low TLR2 activity was not further reduced (+SDS, HF; Fig 1B).

Our previous studies showed that blocking phagocytosis of PGN inhibited the proinflammatory responses of human monocytes [3]. We tested whether the TNFα response of human monocytes to the PGN in the stages of purification were similarly reduced by cytochalasin D. Responses to *B*. *anthracis*-derived PGN at all stages of purification were reduced by cytochalasin D (Fig 1C). Responses to *S*. *aureus*-derived PGN were less sensitive to cytochalasin D but were still reduced (Fig 1D). Thus, responses to the final PGN product from both sources were highest when the material can be internalized by the responding cells but a portion of the response can occur without PGN internalization into the responding cell.

**Fig 2:**
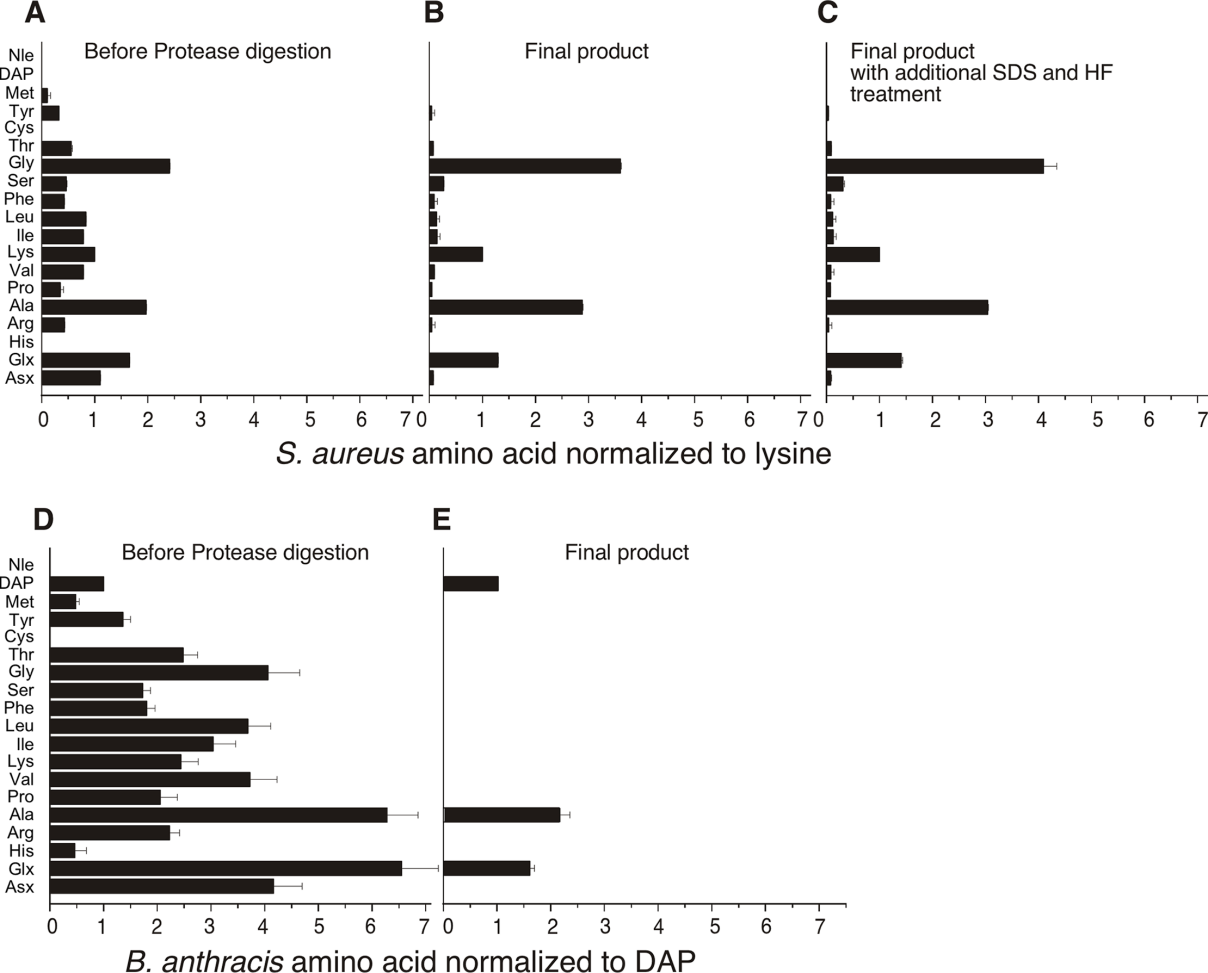
Amino acid analysis of *S*. *aureus and B*. *anthracis* and PGN preparations before and after proteinase K digestion. *S*. *aureus* amino acids were analyzed before (A) and after (B) Proteinase K digestion. The final *S*. *aureus* PGN product was additionally treated with HF, extracted with SDS, and analyzed (C). *B*. *anthracis* amino acids were analyzed before (D) and after (E) proteinase K digestion. Two separate PGN preparations for each species were analyzed, with three replicates of each sample. Data are expressed as the mean ± SEM of amino acid content, normalized to lysine for *S*. *aureus* PGN and to DAP for *B*. *anthracis* PGN.

We analyzed the amino acid content of the PGN preparations before and after proteinase K digestion. Before proteinase K digestion, we found significant levels of a variety of amino acids that are not predicted to be components of the peptide subunit or interpeptide bridges of purified PGN [7]. These amino acids were present in PGN preparations from both pathogens (Fig 2A and 2D). After proteinase K digestion, amino acid complexity was greatly reduced (Fig 2B and 2E), leaving only DAP, Ala, and Glx in the *B*. *anthracis*-derived material (Fig 2E), consistent with the DAP-type PGNs. The *S*. *aureus* PGN had significant levels of Gly, Lys, Ala, and Glx and low levels of other amino acids (Fig 2B). Gly, Lys Ala and Glx are known components of the *S*. *aureus* Lys-type PGN stem peptide and interpeptide bridges [8]. Our analysis was not capable of accurately detecting His or Cys. None of the trace amino acids in the *S*. *aureus* PGN were removed after additional SDS extractions and HF treatment of the final product mentioned above (Fig 2C). Since the two PGN preparations show the predicted amino acid content of the stem peptide and interpeptide bridges, the data indicate that contaminating proteins are absent from the *B*. *anthracis* preparation but traces of some contaminants might be present in the *S*. *aureus* preparation.

### Biological activity is not due to LPS

**Fig 3:**
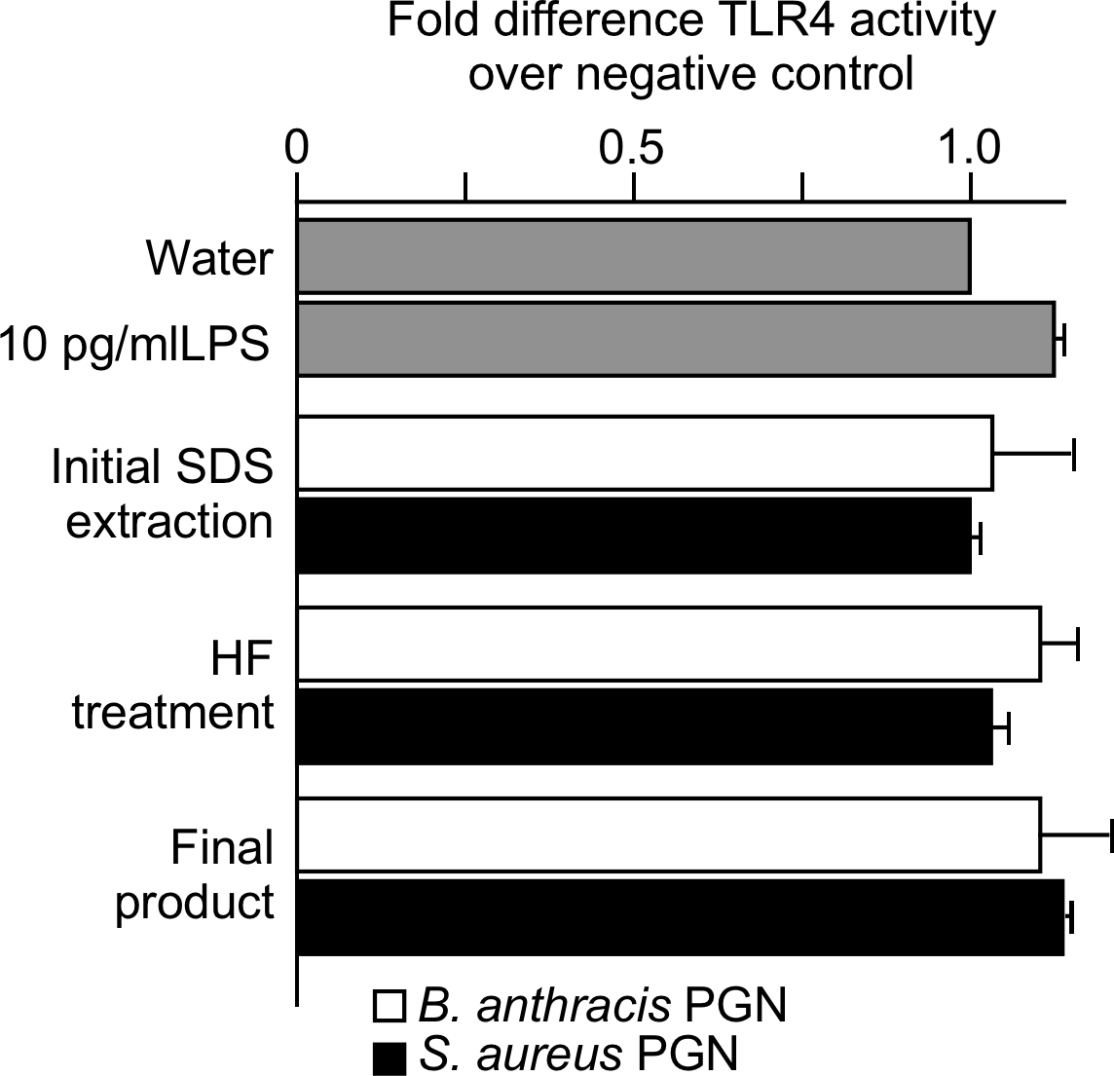
TLR4 activity of *B*. *anthracis* and *S*. *aureus* PGN. *B*. *anthracis* (open bars) and *S*. *aureus* (closed bars) PGN samples were taken after the major steps of the purification process, washed three times with endotoxin free water, dried, weighed, resuspended in water and tested for TLR4 activity at a concentration of 10 μg/ml, using the HEK-TLR4 cell assay. Two separate PGN preparations for each species were tested. The data are expressed as the mean ± SEM fold difference TLR4 activity over the negative control, endotoxin free water. Statistical significance was determined by ANOVA with Bonferroni post test. All PGN samples were not significantly different in TLR4 activity than 10 pg/ml LPS.

The PGN samples were also assayed for LPS using HEK293 cells transfected with human TLR4 and a reporter of NFKB activation. We used endotoxin-free water as a standard and compared all samples to this standard. We also used a dose of 10 pg/ml LPS as a positive control since this amount is detected in this assay and yet the amount is insufficient to stimulate TNFα production from human monocytes [5]. We found (Fig 3) that the LPS content was not significantly different than 10 pg/ml of a commercially prepared endotoxin standard at any stage of the purification process. Thus, the proinflammatory activity of PGN derived from either *B*. *anthracis* or *S*. *aureus* using this protocol and described below and elsewhere is not due to LPS.

### PGN preparations have significantly lower phosphate content than whole bacteria

**Fig 4:**
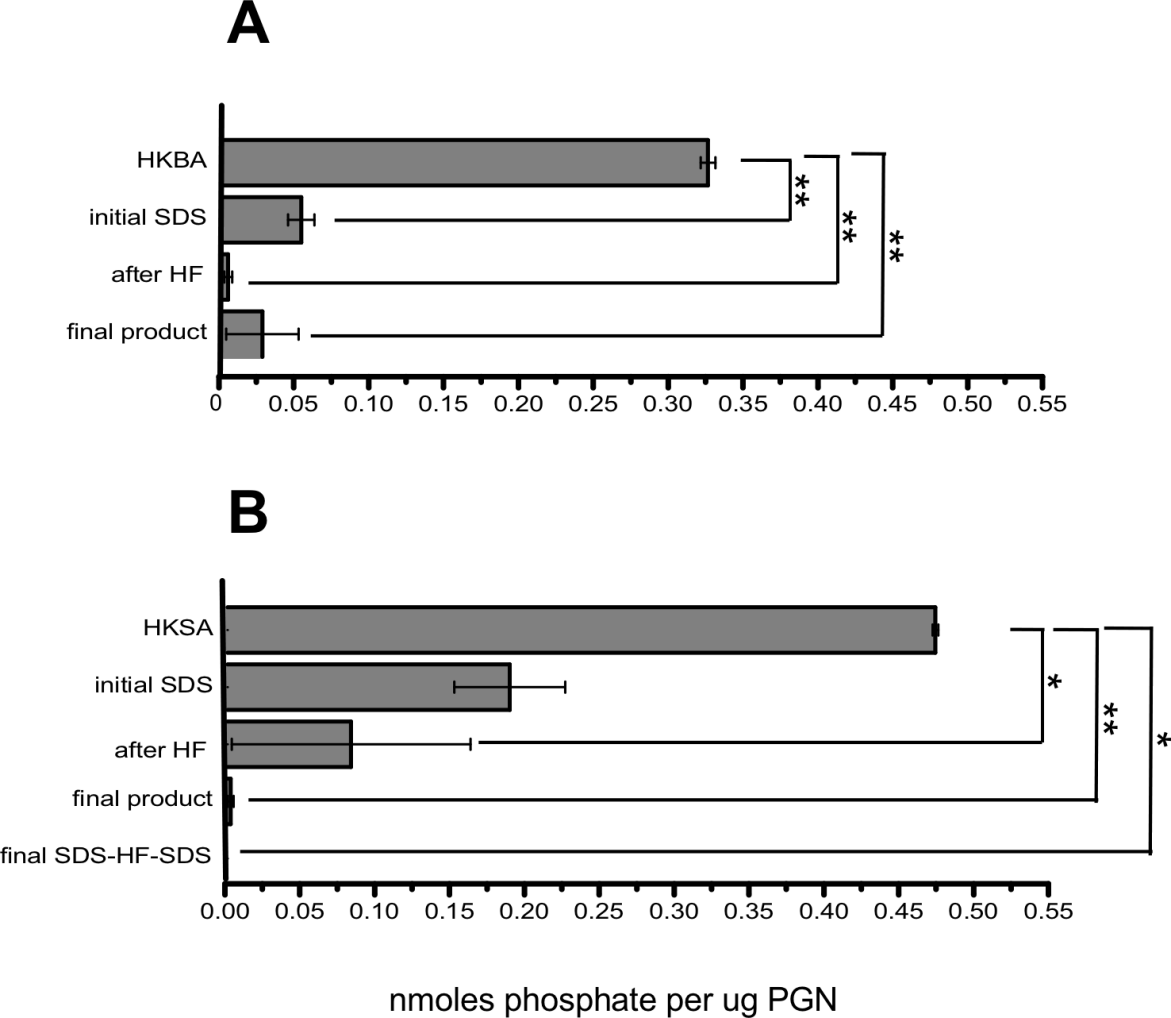
Phosphate content of *B*. *anthracis* and *S*. *aureus* PGN. *B*. *anthracis* (A) and *S*. *aureus* (B) PGN samples were taken after the major steps of the purification process, washed three times with endotoxin free water, dried, weighed, resuspended tested for phosphate content. Two separate PGN preparations for each species were tested, with two replicates for each species. Data are expressed as the mean ± SEM of the nmoles of phosphate per μg/PGN. Statistical significance was determined by ANOVA with Bonferroni post test. *p<0.05, **p<0.01, ***p<0.001 *versus* heat killed bacteria.

We measured the amount of organic phosphate at representative stages of the purification process since TLR2 ligands like lipoteichoic acid have repeating glycerolphosphate units [16]. We subjected samples of PGN and heat-killed bacteria to acid hydrolysis to release phosphate from any organic compound and applied a sensitive measurement of inorganic phosphate to the acid hydrolysate. The results are shown in Fig 4. In order to compare equivalent quantities of PGN, we used a mass of PGN that equals the mass of PGN in the heat killed bacteria. This calculation was based on our determination that 10 X 1^10^ cfu contains 1.25 mg PGN. In the case of *B*. *anthracis*- and *S*. *aureus*-derived PGN, all samples taken from the purification steps showed a significantly reduced phosphate content compared to the corresponding amount present in heat killed bacteria. Thus, heat killed *B*. *anthracis* had 0.3 nmoles phosphate per μg of PGN and heat killed *S*. *aureus* had 0.5 nmoles phosphate per μg of PGN. After the initial SDS extraction, the phosphate content of PGN was reduced to 0.05 nmoles per μg in *B*. *anthracis* PGN and to 0.2 nmoles per μg *S*. *aureus* PGN. After treatment with HF, phosphate content was reduced to 0.004 nmoles/μg for *B*. *anthracis* PGN and to 0.08 nmoles/μg for *S*. *aureus* PGN. Subsequent purification steps, treatment with proteinase K and SDS, did not significantly reduce the phosphate content of either PGN in comparison with the HF treatment (P>0.05). The final PGN products from *B*. *anthracis* and *S*. *aureus* had a phosphate content of 0.03 and 0.002 nmoles phosphate/μg PGN, respectively. These results are consistent with a low level or absence of organic phosphates including forms of teichoic acids that are TLR2 ligands in the PGN final products.

### TLR2 activity in PGN preparations

**Fig 5:**
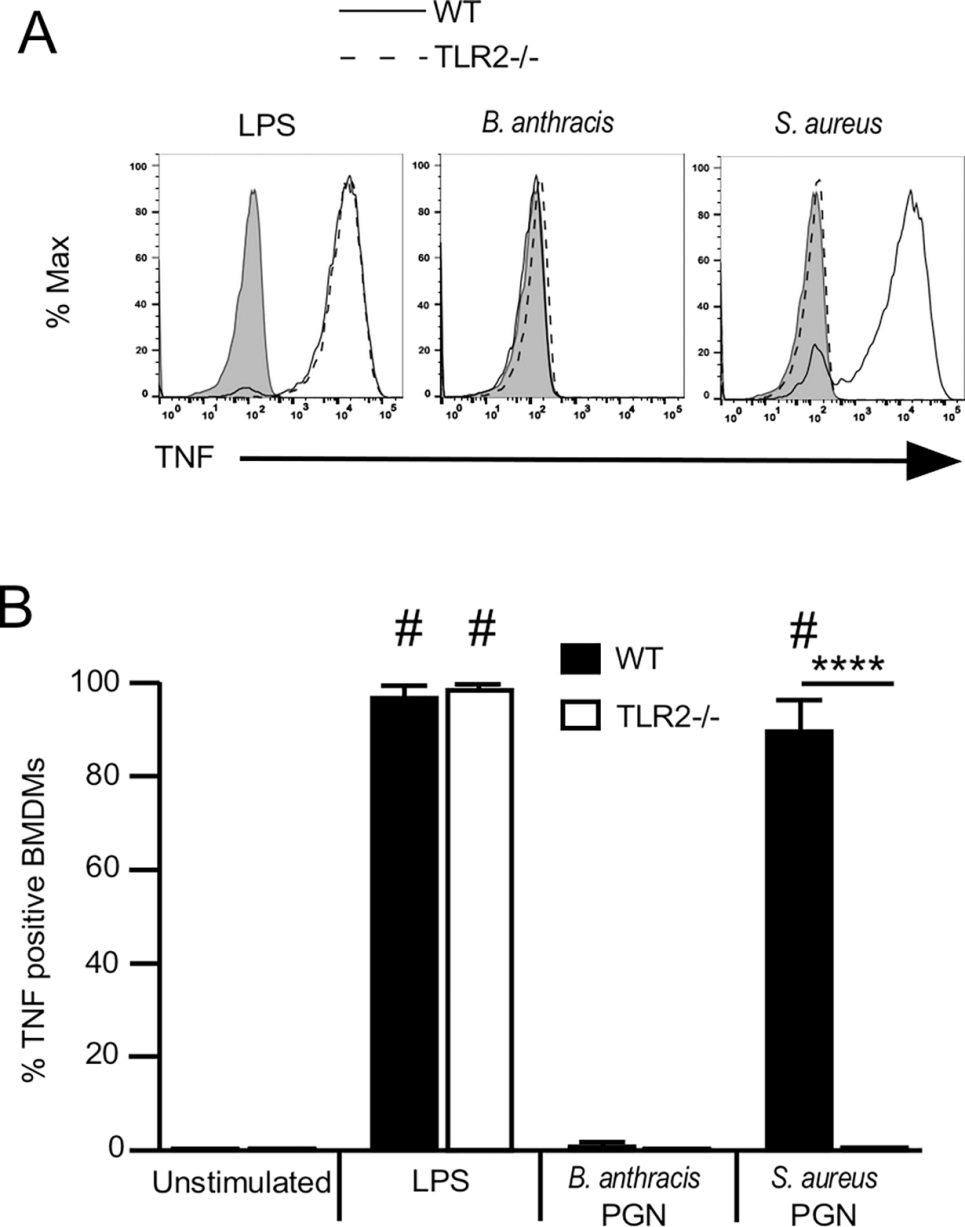
Mouse macrophage response to *B*. *anthracis*- and *S*. *aureus*-derived PGN. (A-B) Differentiated BMDMs from WT or TLR2^-/-^ mice were plated into wells of a 96-well plate (1.5 x 10^5^ cells per well) and stimulated with LPS (1 μg/ml), *B*. *anthracis* PGN (10 μg/ml), or *S*. *aureus* PGN (10 μg/ml) in the presence of Brefeldin A for 6 hours at 37°C. The cells were stained for intracellular TNFα, and then analyzed by flow cytometry. Graphs are representative of 3 independent experiments (solid grey peak is unstimulated sample). Results are mean ± SEM from three independent experiments. The *p* values were calculated using a two-way ANOVA using Bonferroni post hoc test for multiple comparisons. ^#^ *p* < .0001 compared to unstimulated control. **** *p* < .0001 between WT and TLR2^-/-^ BMDMs.

Although we did not detect a response to the PGN preparations using HEK 293 cells expressing sensing elements for TLR2 ligands, we thought mouse bone marrow-derived macrophages might be a more sensitive measure of TLR contaminants. We therefore cultured bone marrow-derived macrophages from wild type and TLR2^-^/^-^ mice with 10 μg/ml *S*. *aureus-* Lys-type PGN or *B*. *anthracis-*derived DAP-type PGN in the presence of brefeldin A, for 6 hours and measured the production of TNFα by intracellular cytokine staining and flow cytometry. The results (Fig 5A) showed that *B*. *anthraci*s-derived PGN failed to stimulate mouse macrophages to make TNFα, consistent with our previous finding that mouse cells do not recognize *B*. *anthracis* PGN [3]. In contrast, *S*. *aureus* PGN produced a significant response (p<0.001) in the wild type mouse macrophages but not in macrophages from TLR2^-/-^ mice. We quantitated the percent responding macrophages in 4 individual experiments and the results are shown in Fig 5B. The ability of Lys-type PGN from *S*. *aureus* to stimulate mouse macrophages in a TLR2-dependent manner suggest that the *S*. *aureus* PGN is itself, or contains, a TLR2 ligand while the *B*. *anthracis*-derived DAP-type PGN is not a TLR2 ligand.

**Fig 6:**
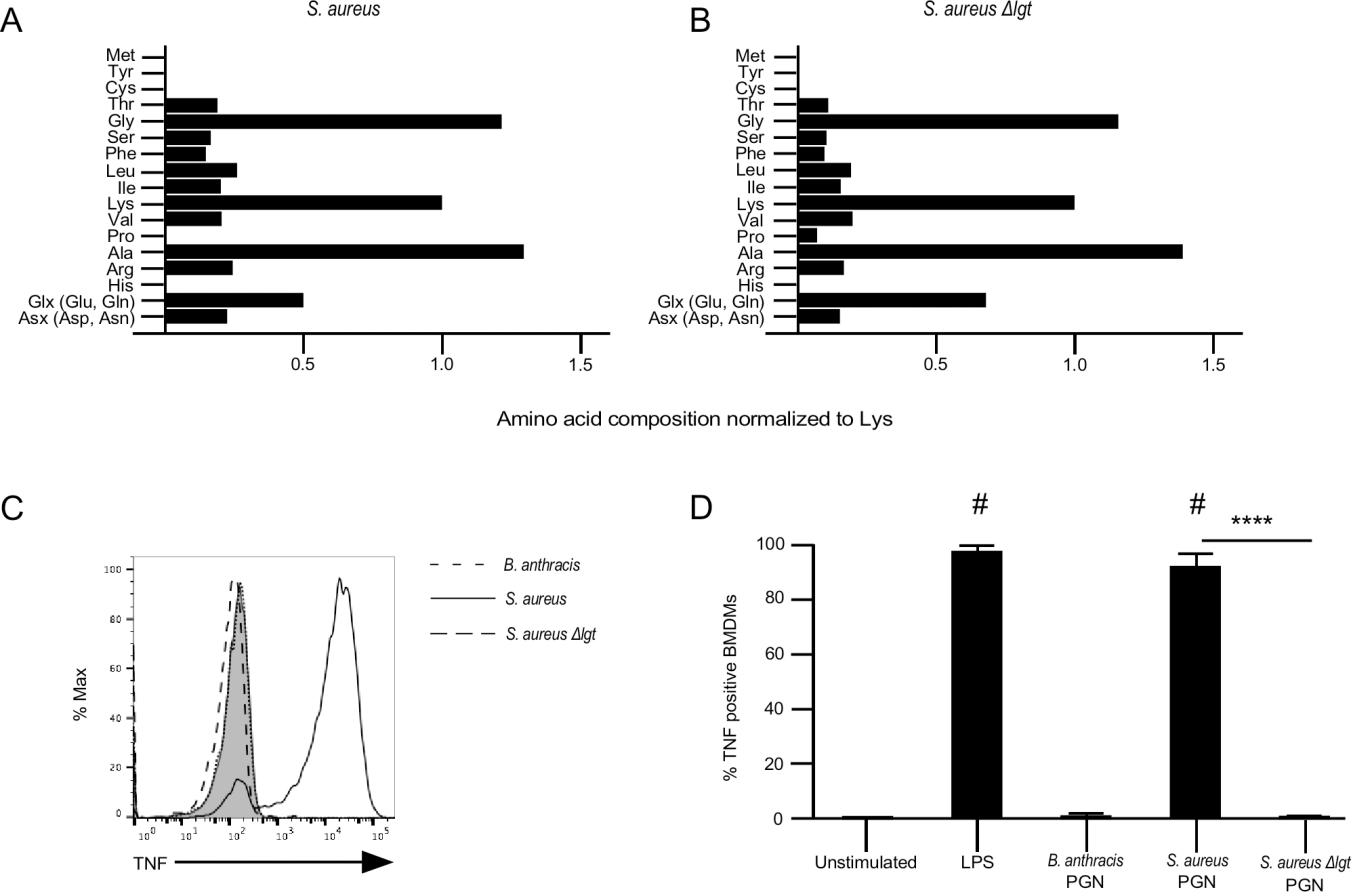
Lipid modification of contaminating peptides in purified *S*. *aureus* PGN is important for TLR2 signaling. PGN from Newman *S*. *aureus* (A) and Newman *S*. *aureus Δlgt* (B) was digested with mutanolysin. After digestion samples were run in triplicate for amino acid analysis as described in Fig 2. (C-D) Differentiated BMDMs were stimulated with LPS (1 μg/ml), *B*. *anthracis* PGN (10 μg/ml), Newman *S*. *aureus* PGN (10 μg/ml), or Newman *S*. *aureus Δlgt* PGN (10 μg/ml) in the presence of Brefeldin A for 6 hours at 37°C. The cells were stained for intracellular TNFα, and then analyzed by flow cytometry. Graphs are representative of three independent experiments (solid grey peak is unstimulated sample). Results are mean ± SEM from three independent experiments. The *p* values were calculated using a one-way ANOVA using Bonferroni post hoc test for multiple comparisons. ^#^ *p* < .05 compared to unstimulated control, ** *p* = .001, **** *p* < .0001.

We noted the amino acid analysis of the *S*. *aureus*-derived PGN showed trace amounts of amino acids that cannot be attributed to the stem peptide (Fig 2). These trace amino acids may be lipidated peptides that fail to be removed by the protease K digestions during purification. Accordingly, the PGN from *S*. *aureus* might be contaminated with TLR2 ligands. To test this possibility, we purified PGN using the same procedure described above and applied to the *S*. *aureus* strain Newman and its isogenic mutant lacking lipoprotein diacylglycerol transferase (Δlgt) [17]. The PGN products from these two strains were tested on mouse bone marrow-derived macrophages. We found (Fig 6A and 6B) that the remaining amino acid impurities are present in approximately equal amounts in the PGN material derived from either the parent or the Δ*lgt* mutant *S*. *aureus* strains. We applied our flow cytometry-based assay to measure TNFα production and found that only PGN derived from the parent strain having the lipoprotein transferase was able to stimulate mouse bone marrow-derived macrophages (Fig 6C); PGN derived from the Δlgt *S*. *aureus* mutant failed to stimulate, as did PGN derived from *B*. *anthracis*. The average and standard error of three separate experiments are shown in Fig 6D. This finding indicates that the material in the PGN that stimulates mouse macrophages is the contaminating lipopeptides and not PGN itself. The data are consistent with the result in Fig 5, showing that PGN derived from *B*. *anthracis* is not recognized by mouse macrophages. Taken together with the results shown in Fig 6, we conclude that PGN itself, regardless of the bacterial species of origin, is not a TLR agonist.

**Fig 7:**
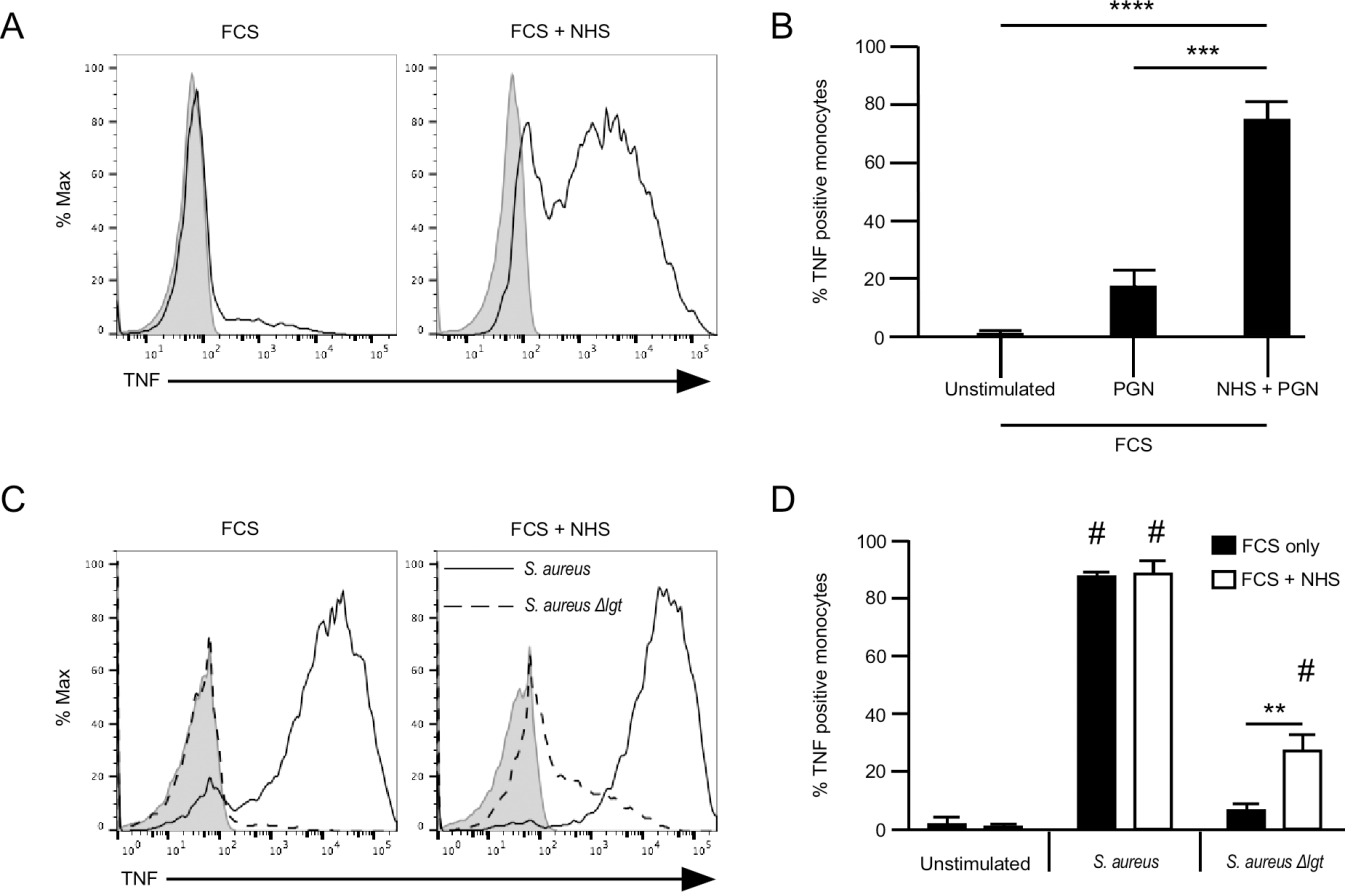
Human monocyte responses to PGN from lipoprotein deficient *S*. *aureus* require serum opsonins. (A) PBMCs were plated into wells of a 96-well plated (4–8 x 10^5^ cells per well) and stimulated with *B*. *anthracis* PGN (10 μg/ml) in presence of FCS (1% v/v) with or without normal human serum (1% v/v) and with Brefeldin A for 6 hours at 37°C. The cells were stained for intracellular TNFα, and then analyzed by flow cytometry. (B) PBMCs were plated into wells of a 96-well plated (4–8 x 10^5^ cells per well) and stimulated with Newman *S*. *aureus* PGN (10 μg/ml), or Newman *S*. *aureus Δlgt* PGN (10 μg/ml) in presence of FCS (1% v/v) with or without normal human serum (1% v/v) and with Brefeldin A for 6 hours at 37°C. The cells were stained for intracellular TNFα, and then analyzed by flow cytometry. Graphs are representative of experiments from three donors (solid grey peak is unstimulated sample). Results are mean ± SEM from three donors. The *p* values were calculated using either a two-way (B) or one-way (D) ANOVA using Bonferroni post hoc test for multiple comparisons. ^#^ *p* ≤ .0001 compared to unstimulated control, ** *p* = .001, ***p = 0001, **** *p* < .0001.

Earlier, we showed the human responses to purified PGN is elevated by the presence of serum factors like IgG which opsonize PGN and enhance its uptake into phagocytic cells [3, 13]. Thus, we can use the enhancement effect of human serum to test the purity of the various PGN preparations. The flow cytometry-based measurements of TNFα are shown in the left two panels of Fig 7 and a summary of three experiments is shown in the right two panels of Fig 7. *B*. *anthracis*-derived PGN was a weak agonist in the absence of human serum and a potent agonist in the presence of human serum (Fig 7A and 7B). In contrast, PGN derived from the *S*. *aureus* Newman strain having the lipoprotein transferase activity was a potent agonist towards human monocytes regardless of the presence or absence of human serum (Fig 7C and 7D). PGN derived from the Δlgt mutant failed to stimulate human monocytes in the absence of serum and was a weak agonist in the presence of serum. These results are consistent with those shown in Fig 6: the contaminating lipopeptides are able to stimulate human monocytes in a human serum-independent way, while PGN lacking the lipopeptides requires human serum to stimulate the cells.

## Discussion

We have performed a series of experiments to determine whether the DAP-type *B*. *anthracis* and/or Lys-type *S*. *aureus* PGNs are activating TLR2 ligands and/or to determine at what point in the purification process activating TLR ligands are removed. We found, in the case of both bacterial types, that heat killed bacteria stimulate HEK293 cells strongly through TLR2, indicating that TLR2 ligands are present in the unprocessed bacteria. However, we found differences in the amount of TLR2 activity retained during purification of PGN from these bacteria. For *B*. *anthracis*, the PGN signaling through TLR2 was lost after the initial extraction from bacterial cultures with boiling SDS. This finding suggests that TLR2 ligands might be embedded in the PGN layer of whole bacteria but are easily removed by the detergent. The TLR2 activity remained below the level of significance throughout the extraction process, except that there was a small increase through the addition of an exogenous processing agent, proteinase K. This TLR2 activity was easily removed by additional SDS extraction and washing to produce a TLR2 ligand-free final product. In contrast, for the *S*. *aureus* PGN, significant TLR2-stimulating activity was present after the initial SDS extraction as detected by HEK293-TLR2 cells and mouse bone marrow-derived macrophages. The TLR2 activity was reduced after HF treatment but largely remained throughout processing. Additional HF treatment, SDS extractions and washing did not further remove the TLR2 activity. Lastly, we showed that the TLR2 activity in *S*. *aureus*-derived PGN was not PGN itself but caused by contaminating lipopeptides. We conclude that neither DAP-type *B*. *anthracis* PGN nor Lys-type *S*. *aureus* PGN is a TLR2 ligand. However, it is clear that insufficient purification of PGN could lead to introduction of or contamination by existing TLR2 ligands. Insufficient PGN purification may have been a factor in past studies leading to the notion that PGN is a TLR2 ligand. We were unable to remove the TLR2 activity from PGN derived from *S*. *aureus* except from genetically manipulated strains.

Our data also show that mouse bone marrow-derived macrophages are a more sensitive indicator of TLR2 activity than are HEK293 transfected with the TLR2 receptor and a NF-kB reporter system. Mouse macrophages produced a significant inflammatory response to *S*. *aureus* PGN through TLR2 activation (Fig 3), whereas the HEK293 cells detected a very low and statistically insignificant level of TLR2 activity in the identical sample (Fig 1).

The response of human monocytes to the Lys-type PGN from *S*. *aureus* Δ*lgt* mutant was considerably weaker in the presence of human serum compared to the response to *B*. *anthracis*-derived PGN. We attribute this difference to the fact that *B*. *anthracis*-derived PGN has DAP-containing stem peptides that are lacking in *S*. *aureus* PGN. After lysosomal digestion, *S*. *aureus* PGN can form the NOD2 ligand muramyl dipeptide to stimulate innate immune cells [18]. Likewise, after lysosomal digestion, *B*. *anthracis* PGN can form muramyl dipeptide and several DAP-containing peptides that are also ligands for NOD1 [19]. Activation of both NOD1 and NOD2 by *B*. *anthracis* PGN might account for the relative potency of these forms of PGN to stimulate TNFα production by monocytes.

The poly-disaccharide nature of PGN does not chemically resemble other known TLR2 ligands. Natural TLR2 ligands each have a hydrophobic component, such as present in glycolipids or lipopeptides [16, 20]. Other TLR2 ligands are amphiphilic such as LTA [16, 21], lipoarabinomannan [22] and glycosylphosphatidylinositol-anchored lipids [23]. However, PGN, as a poly-disaccharide linked by largely polar amino acids, is not hydrophobic. This has led to questions about TLR2’s ability to recognize such a chemically diverse set of PAMPs while still maintaining the sensitivity expected of a PRR of the innate immune system [16, 24]. Our study explains that TLR2 does not recognize the PGN poly-disaccharide and so the recognition of diverse PAMPs by TLR2 may no longer be a relevant inconsistency.

We have used the need for phagocytosis of PGN as a criterion for its purity. Although most of the proinflammatory response is sensitive to agents that block phagocytosis like cytochalasin D, not all of the response is blocked. It may be that some of the PGN particles can enter cells using a process that is not sensitive to agents that disrupt actin reorganization like cytochalasin D. However, it may be that PGN is able to weakly activate immune cells through a surface receptor that is as yet unidentified.

We have also used the lack of a response by mouse macrophages a criterion for PGN purification. It is unclear why mouse cells fail to respond to PGN with inflammatory cytokine production. Earlier, we showed that mice lack the PGN serum opsonins like anti-PGN antibodies that are present in humans [13]. The lack of these opsonins could prevent mouse macrophages from taking up and digesting PGN to form ligands that can be sensed by cytoplasmic NOD receptors. However, in unpublished studies, we found that experimentally-generated mouse anti-PGN IgG fails to support a response by mouse macrophages to *B*. *anthracis*-derived PGN. Likewise, we found that human IgG containing anti-PGN IgG does not support an inflammatory response in macrophages derived from mice that express human FcγRs as transgenes [25]. Mouse macrophages have been reported to respond to PGN but the activation in these reports require that the macrophages are first primed with agents like LPS [see, e.g., [26]]. Priming with such agents could alter macrophage phagocytic ability, the need for serum opsonins, or NOD expression [27]. Mice lacking NOD1 or NOD2 show a phenotype when challenged with bacterial pathogens [reviewed in [28]], including *B*. *anthracis* [29, 30], suggesting that the PGN is recognized in some way. In these examples using live bacterial challenges in NOD-deficient animals, it may be that other bacterial PAMPS serve the priming function and allow PGN to be taken up in ways distinct from human cells. However, it may also be that some of the inflammation and accompanying pathology caused by bacterial pathogens which occurs in humans is not represented in mouse models because of the lack of PGN responses that are similar to humans.

## Abbreviations

APC: allophycocyanin
ANOVA: analysis
Ba: *Bacillus anthracis*
BMDM: bone marrow-derived macrophages
CSF-1: colony stimulation factor-1
DAP: diaminopimelic acid
LAL: endotoxin free
FBS: fetal bovine sera
HK: heat-killed
HEK-TLR2: HEK293 cells stably expressing human TLR2 and CD14
HEK-TLR4: HEK293 cells stably expressing human TLR4 and MD-2/CD14
HF: hydrofluoric acid
FcγR: IgG/Fc receptors
LPS: lipopolysaccharide
lgt: lipoprotein diacylglycerol transferase
LTA: lipoteichoic acids
PAMP: pathogen-associated molecular pattern
PRR: pattern recognition receptor
PGN: peptidoglycan
PBMC: peripheral blood mononuclear cells
SEAP: secreted embryonic alkaline phosphatase
Sa: *Staphylococcus aureus*
TLR2: Toll-like receptor 2
WTA: wall teichoic acids
WT: wild type
